# Value of [^68^Ga]Ga-NYM046 PET/CT, in Comparison with ^18^F-FDG PET/CT, for Diagnosis of Clear Cell Renal Cell Carcinoma

**DOI:** 10.2967/jnumed.124.267527

**Published:** 2024-12

**Authors:** Kequan Lou, Jialiang Wang, Huihui He, Yanjuan Wang, Yuanyuan Mi, Wenjin Li, Liping Chen, Yu Zhang, Yong Mao, Jianguo Lin, Haitian Fu, Chunjing Yu

**Affiliations:** 1 Department of Nuclear Medicine, Affiliated Hospital of Jiangnan University, Wuxi, China;; 2 Department of Urological Surgery, Affiliated Hospital of Jiangnan University, Wuxi, China;; 3 Wuxi School of Medicine, Jiangnan University, Wuxi, China;; 4 Department of Oncology, Affiliated Hospital of Jiangnan University, Wuxi, China; and; 5 Jiangsu Key Laboratory of Molecular Nuclear Medicine, Jiangsu Institute of Nuclear Medicine, Wuxi, China

**Keywords:** [^68^Ga]Ga-NYM046, ^18^F-FDG, PET/CT, carbonic anhydrase IX, clear cell renal cell carcinoma

## Abstract

This study aimed to investigate the diagnostic efficacy of [^68^Ga]Ga-NYM046 PET/CT in animal models and patients with clear cell renal cell carcinoma (ccRCC) and to compare its performance with that of ^18^F-FDG PET/CT. **Methods:** The in vivo biodistribution of [^68^Ga]Ga-NYM046 was evaluated in mice bearing OS-RC-2 xenografts. Twelve patients with ccRCC were included in the study; all completed paired [^68^Ga]Ga-NYM046 PET/CT and ^18^F-FDG PET/CT. The diagnostic efficacies of these 2 PET tracers were compared. Moreover, the positive rate of carbonic anhydrase IX in the pathologic tissue sections was compared with the SUV_max_ obtained by PET/CT. **Results:** The tumor accumulation of [^68^Ga]Ga-NYM046 at 1 h after injection in OS-RC-2 xenograft tumor models was 7.21 ± 2.39 injected dose per gram of tissue. Apart from tumors, the kidney and stomach showed high-uptake distributions. In total, 9 primary tumors, 96 involved lymph nodes, and 147 distant metastases in 12 patients were evaluated using [^68^Ga]Ga-NYM046 and ^18^F-FDG PET/CT. Compared with ^18^F-FDG PET/CT, [^68^Ga]Ga-NYM046 PET/CT detected more primary tumors (9 vs. 1), involved lymph nodes (95 vs. 92), and distant metastases (137 vs. 127). In quantitative analysis, the primary tumors’ SUV_max_ (median, 13.5 vs. 2.4; *z* = −2.668, *P* = 0.008) was significantly higher in [^68^Ga]Ga-NYM046 PET/CT. Conversely, the involved lymph nodes’ SUV_max_ (median, 5.9 vs. 7.6; *z* = −3.236, *P* = 0.001) was higher in ^18^F-FDG PET/CT. No significant differences were found for distant metastases (median SUV_max_, 5.0 vs. 5.0; *z* = −0.381, *P* = 0.703). Higher [^68^Ga]Ga-NYM046 uptake in primary tumors corresponded to higher expression of carbonic anhydrase IX, with an *R*^2^ value of 0.8274. **Conclusion:** [^68^Ga]Ga-NYM046 PET/CT offers a viable strategy for detecting primary tumors, involved lymph nodes, and distant metastases in patients with ccRCC.

## Visual Abstract

**Figure:**
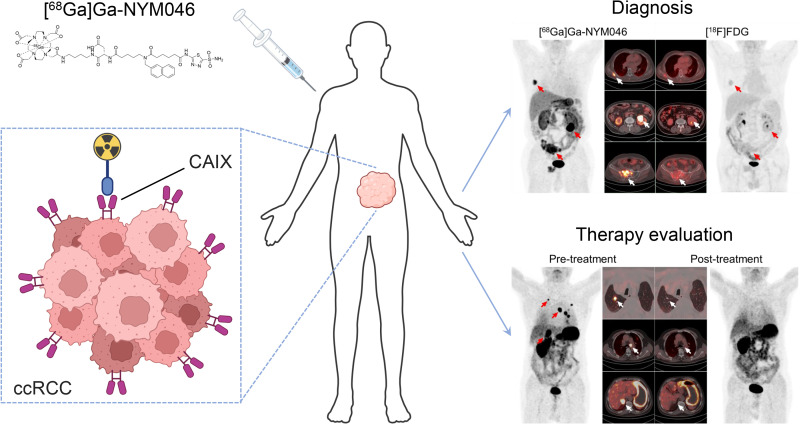

Renal malignancies account for 2% of the annual global tumor incidence, with clear cell renal cell carcinoma (ccRCC) being predominant (1). The success of treating patients depends on early diagnosis and accurate staging, which are crucial in guiding personalized therapeutic management (2,3). About 20%–30% of individuals who have undergone surgery for renal cell carcinoma are at risk of metastatic relapse (4). Such relapses can manifest in unusual sites such as the pancreas, peritoneal cavity, and intestinal tract, which are likely to be missed by routine follow-up (5,6). ^18^F-FDG PET/CT is a commonly used method for whole-body evaluation of tumors. Nevertheless, its performance in diagnosing primary foci of ccRCC has been disappointing, as previous metaanalyses demonstrated a sensitivity of only 62% (7). In addition, some studies revealed that ^18^F-FDG PET/CT faces inherent challenges in diagnosing metastatic lesions of ccRCC because of the high physiologic background activity and inflammation, with a variable sensitivity range of 63.6%–90.0% (8–10). Consequently, these limitations underscore the need to develop alternative diagnostic strategies to improve the accuracy of tumor detection.

Carbonic anhydrase IX (CAIX) belongs to the phylogenetically well-preserved carbonic anhydrase family and is induced by hypoxia, functionally related to the acidic tumor microenvironment, involved in tumor aggressiveness (11,12). Moreover, CAIX is highly expressed in nearly all cases of ccRCC, triggered by the inactivation of von Hippel–Lindau syndrome (13). By contrast, CAIX expression in adult normal tissues is relatively low, except in the stomach, bile duct epithelium, and gallbladder (14). A CAIX-targeting mouse IgG1 monoclonal antibody (known as G250 or girentuximab) was first labeled with ^131^I and applied as an imaging agent in patients with ccRCC (15). Subsequently, ^111^In- or ^89^Zr-girentuximab entered clinical research (16–19). However, the findings from previous studies indicated that large-molecule antibodies often exhibit limited tumor penetration and slow blood clearance. Compared with large-molecule biopharmaceuticals, small-molecule targeted drugs exhibit superior characteristics in various domains, including pharmacokinetic behavior, cost, patient adherence, and ease of drug storage and transportation (20).

Here, a new small-molecule compound, NYM046, targeting CAIX was introduced. It is based on acetazolamide and features a DOTA chelating structure for ^68^Ga labeling. In this study, the diagnostic efficacy of [^68^Ga]Ga-NYM046 in OS-RC-2 xenograft tumor models and patients with ccRCC was evaluated, and its application value was compared with that of ^18^F-FDG PET/CT.

## MATERIALS AND METHODS

### Radiopharmaceutical Preparation

NYM046 was synthesized by Shanghai Bioduro Biologics Co., Ltd., by the authors’ design. Radionuclide labeling was performed using the Morten M1 system (Sunmao Medical Technologies). In brief, ^68^Ga was prepared using a ^68^Ge/^68^Ga generator (Eckert & Ziegler) and 0.05 M HCl (5 mL). The eluate was added to a reaction flask containing a sodium acetate buffer (0.25 M), along with 40 μg (0.036 μmol) of the precursor, NYM046 (1 mg/mL), to achieve a mixture with a pH of 4. The mixture was heated to 100°C for 10 min and purified using a C18 Sep-Pak cartridge (Waters), with 0.5 mL of 75% ethanol solution and 5 mL of normal saline as eluents. The product, [^68^Ga]Ga-NYM046, was confirmed by radioactive high-performance liquid chromatography (Thermo Fisher Scientific, Inc.).

### Small-Animal PET/CT and Blocking Study

Tumor-bearing mice were anesthetized by administration of 1.5%–2% isoflurane through an air current set at 0.5 L/min and then were intravenously injected with [^68^Ga]Ga-NYM046 (2.3 ± 0.1 MBq, 0.2 mL). Dynamic scanning using small-animal PET/CT followed (Super Nova) within 3 h. Small-animal PET/CT images were reconstructed using 3-dimensional ordered-subsets expectation maximization. Then, the major organs were manually outlined on the images as the region of interest. The percentage injected dose per gram of tissue (%ID/g) for major organs was calculated using PMOD (version 4.3; PMOD Technologies). For blocking experiments, tumor-bearing mice were administered unlabeled NYM046 (400 μg, 0.2 mL) intravenously, followed 1 h later by an intravenous injection of [^68^Ga]Ga-NYM046 (2.1 ± 0.1 MBq, 0.2 mL), with scanning performed at 1 h after injection. Animal experiments were performed in compliance with the guidelines established by the ethical committee of Jiangnan University.

### Patient Inclusion Criteria

This study was a prospective, single-center trial to evaluate the diagnostic performance of [^68^Ga]Ga-NYM046 PET/CT in patients with primary or metastatic ccRCC. Twelve patients were ultimately enrolled, all of whom had undergone surgery or puncture biopsy of renal lesions with a confirmed diagnosis of ccRCC (Supplemental Fig. 1; supplemental materials are available at http://jnm.snmjournals.org). The study was authorized by the Ethics Committee of the Jiangnan University affiliated hospital (LS2020003). All patients provided written informed consent to participate in the study. The study was registered at ClinicalTrials.gov (NCT05638256).

### PET/CT in Patients

Each patient underwent paired [^68^Ga]Ga-NYM046 and ^18^F-FDG imaging using a PET/CT scanner (Siemens Biography 64 TruePoint). The interval between the 2 scans was less than 3 d. Scans were taken from the head to the upper femur and divided into separate head and body portions. The mean dose of [^68^Ga]Ga-NYM046 was 129 MBq (range, 98–174 MBq) for each patient, whereas the dose of ^18^F-FDG was based on the patient’s weight (5.55 MBq/kg).

### PET/CT Image Analysis

[^68^Ga]Ga-NYM046 and ^18^F-FDG PET/CT images were viewed independently by 2 qualified nuclear medicine physicians. Any lesion with higher radioactivity uptake than in the surrounding normal tissue was considered positive (except for physiologic uptake). The short diameters of involved lymph nodes and the long diameters of other measurable lesions were measured according to the RECIST 1.1 standard. SUV_max_ was obtained by outlining the region of interest.

### Statistical Analysis

All statistical analyses were accomplished using SPSS 25.0 (IBM). SUV_max_ data were expressed as median, first quartile, and third quartile and were compared using the nonparametric Wilcoxon signed-rank test. Lesion numbers were assessed using the McNemar test. Statistical differences were considered significant if the *P* value was less than 0.05.

## RESULTS

### Chemistry and Radiolabeling

**FIGURE 1.**
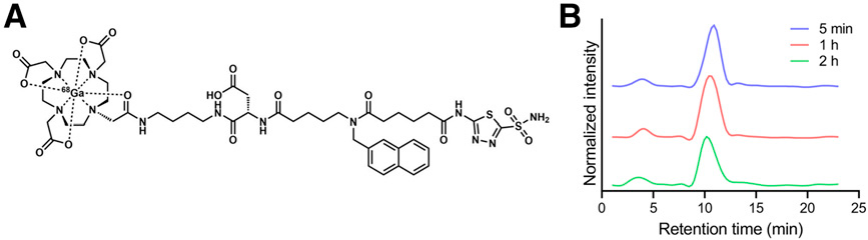
(A) Chemical structure of [^68^Ga]Ga-NYM046. (B) Stability of [^68^Ga]Ga-NYM046 in serum on radioactive high-performance liquid chromatography analysis.

The chemical structure and characterization of NYM046 are shown in Supplemental Fig. 2. NYM046 exhibited a molecular mass of 1,118.45 g/mol and a purity of 95.74% (Supplemental Fig. 2). The chemical structure of [^68^Ga]Ga-NYM046 is shown in Figure 1A, and its molar activity was 3.68 ± 0.83 GBq/μmol. The radiochemical purity of [^68^Ga]Ga-NYM046 was more than 95% (Supplemental Fig. 3). Additionally, [^68^Ga]Ga-NYM046 remained stable for more than 2 h in human serum (Fig. 1B). The binding affinity of [^68^Ga]Ga-NYM046 to CAIX was 4.98 ± 0.50 nM (Supplemental Fig. 4). During the safety assessment, 6 mice were injected with [^68^Ga]Ga-NYM046 (7.4 MBq, 0.5 mL) via the tail vein, and no body weight loss was observed over 14 d (Supplemental Fig. 5).

### PET/CT in Animals

**FIGURE 2.**
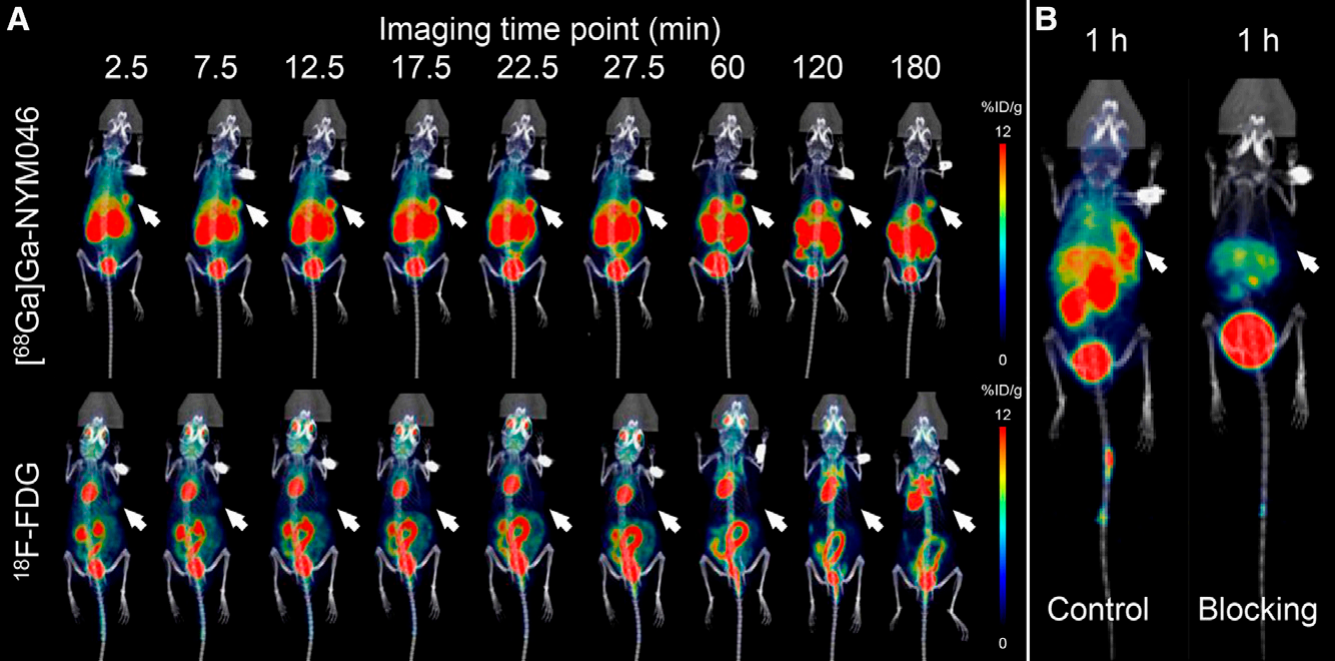
(A) In vivo biodistribution imaging of [^68^Ga]Ga-NYM046 and ^18^F-FDG in OS-RC-2 xenograft tumor models. (B) Representative PET/CT images of OS-RC-2 xenograft tumor models with or without unlabeled NYM046 blocking.

The radioactivity uptake of [^68^Ga]Ga-NYM046 in OS-RC-2 xenograft tumor models peaked between 27.5 and 60 min (Fig. 2A), and the tumor accumulation at 1 h after injection was 7.21 ± 2.39 %ID/g (Supplemental Table 1). The blockade study of [^68^Ga]Ga-NYM046 showed a decrease in tumor radioactivity uptake from 8.66 ± 2.17 %ID/g to 2.46 ± 0.67 %ID/g for 1 h (Fig. 2B).

**FIGURE 3.**
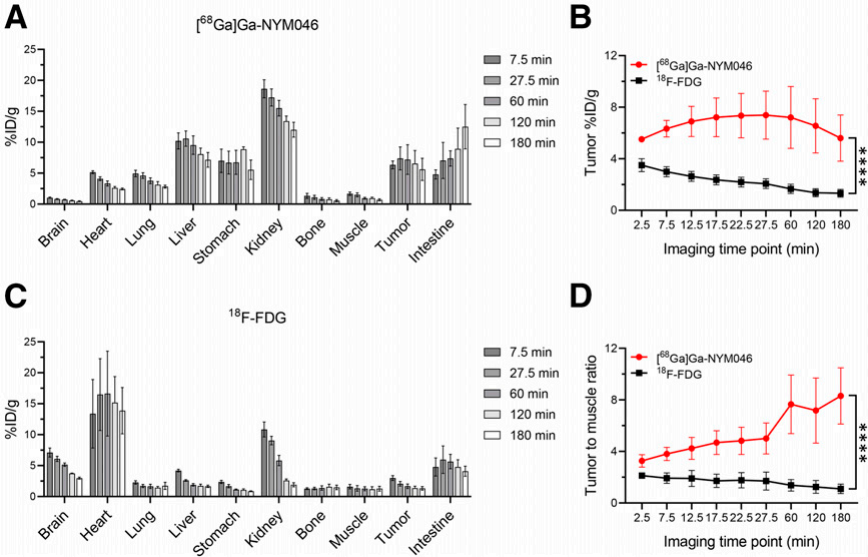
(A and C) Radioactivity biodistribution of [^68^Ga]Ga-NYM046 (A) and ^18^F-FDG (C) in various organs in OS-RC-2 tumor model at different time points after injection. (B and D) Quantitative curves of tumor uptake (B) and contrast (D) with background tissues (tumor-to-muscle contrast). ****P < 0.0001.

The radioactivity biodistributions of [^68^Ga]Ga-NYM046 and ^18^F-FDG in the OS-RC-2 xenograft tumor models was assessed (Figs. 3A and 3C). The tumor accumulation and tumor-to-muscle ratio were significantly higher for [^68^Ga]Ga-NYM046 PET/CT than for ^18^F-FDG PET/CT within 3 h (Figs. 3B and 3D).

### Clinical PET/CT Studies in Patients with ccRCC

Patient clinical data are presented in Table 1. Twelve patients (9 male and 3 female; median age, 59 y; range, 38–81 y) who underwent paired [^68^Ga]Ga-NYM046 and ^18^F-FDG PET/CT were prospectively enrolled. No adverse reactions were observed in patients after [^68^Ga]Ga-NYM046 PET/CT in a 2-wk follow-up period.

**TABLE 1.** Characteristics of Included Clinical Cases

Patient no.						SUV_max_	
	Sex	Age (y)	Condition	Tumor location	Lesions*	[^68^Ga]Ga-NYM046	^18^F-FDG
1	F	56	Preop	Primary tumor	1/0	8.2	1.9
2	F	46	Preop	Primary tumor	1/0	6.2	2.5
3	F	54	Postop	Pleura, pancreas mets	7/0	4.3 (2.6, 8.9)	2.0 (1.5, 2.4)
4	M	67	Preop	Primary tumor	1/0	3.7	2.3
5	M	63	Preop	Primary tumor	1/0	13.5	2.9
6	M	38	Preop	Primary tumor	1/0	11.5	2.6
7	M	59	Preop	Primary tumor	1/0	13.9	2.2
				Lymph node met	0/1	5.0	1.6
				Lung and bone mets	3/1	6.5 (4.1, 13.8)	2.4 (1.4, 3.0)
8	M	46	Preop	Primary tumor	1/0	13.9	2.2
9	M	72	Preop	Primary tumor	1/0	18.5	2.6
10	M	59	Postop	Lymph node mets	19/72	5.8 (4.7, 7.5)	7.8 (5.6, 9.4)
				Brain, lung, liver, bone and subcutaneous mets	55/68	4.9 (3.7, 6.5)	5.5 (4.2, 6.9)
11	M	81	Preop	Primary tumor	1/0	43.7	2.4
				Lung, adrenal gland and bone mets	5/4	11.2 (5.3, 15.3)	2.8 (1.8, 4.0)
12	M	73	Postop	Lymph node mets	3/1	21.4 (9.6, 24.9)	3.6 (2.5, 4.0)
				Lung and bone mets	2/2	13.1 (7.9, 16.9)	2.0 (1.0, 9.0)

* Measurable/nonmeasurable lesions on CT according to RESIST 1.1 standard.

Preop = before operation; postop = after operation; met = metastasis.

SUV_max_ is expressed as specific numeric value or as median followed by first and third quartiles in parentheses.

**FIGURE 4.**
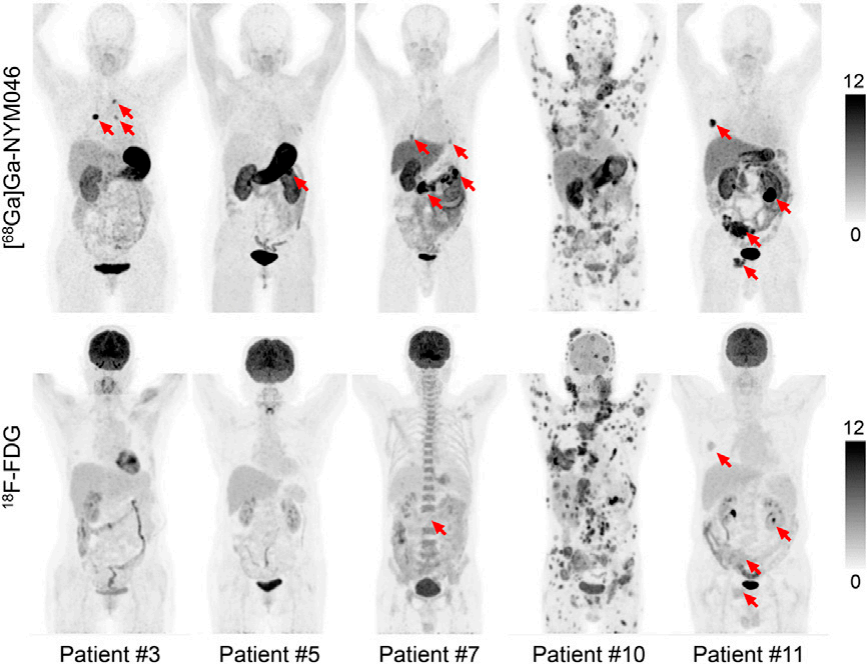
Representative maximum-intensity projections of 5 patients (patients 3, 5, 7, 10, and 11) comparing [^68^Ga]Ga-NYM046 and ^18^F-FDG. Both tracers showed specific retention in bone and lymph node metastases (patients 10 and 11). [^68^Ga]Ga-NYM046 PET/CT outperformed ^18^F-FDG PET/CT in detecting primary tumors, lung metastases, and pleural metastases (patients 3, 5, 7, and 11).

Representative PET/CT images of 5 patients at 1 h after injection of [^68^Ga]Ga-NYM046 and ^18^F-FDG are shown in Figure 4. The tissues with the highest SUV_max_ uptake of [^68^Ga]Ga-NYM046 were the stomach (22.14 ± 16.40), tumor (8.60 ± 5.98), kidney (6.92 ± 2.24), and gallbladder (6.68 ± 2.04; Supplemental Table 2). The SUV_max_ of ^18^F-FDG in tumor was 1.87 ± 0.48, which was lower than that of [^68^Ga]Ga-NYM046 (Supplemental Table 2).

**FIGURE 5.**
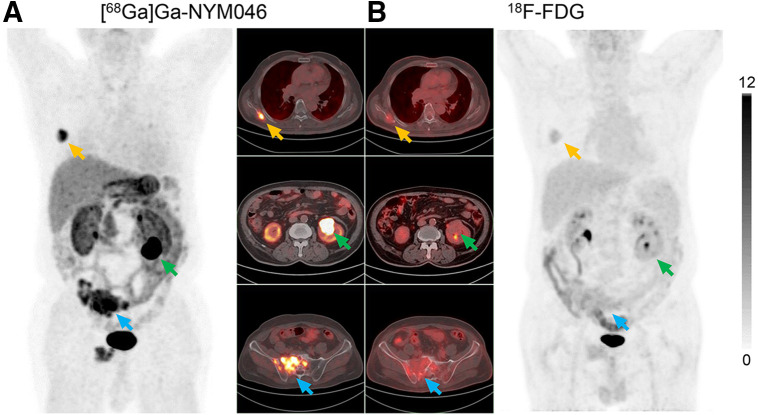
[^68^Ga]Ga-NYM046 PET/CT (A) and ^18^F-FDG PET/CT (B) scans of patient 11. Bone metastases (yellow and blue arrows) were diagnosed as positive findings in both scans, whereas renal primary tumor (green arrows) was diagnosed as positive finding on [^68^Ga]Ga-NYM046 PET/CT but as false-negative finding on ^18^F-FDG PET/CT.

In visual analysis, the comparison revealed that [^68^Ga]Ga-NYM046 PET/CT detected more primary tumors (9/9 vs. 1/9, *P* = 0.008) and distant metastases (137/147 vs. 127/147, *P* = 0.041). Meanwhile, these 2 scans had roughly similar efficacy in diagnosing lymph node metastases (Table 2). A typical image comparing the 2 scans is shown (Fig. 5). Additionally, 103 of 252 CT-measurable lesions were positive for [^68^Ga]Ga-NYM046 uptake, or approximately 41%.

**TABLE 2.** Comparison of [^68^Ga]Ga-NYM046 and ^18^F-FDG PET/CT in Patients at 1 Hour After Injection

	SUV_max_				Lesions (*n*)			
Parameter	[^68^Ga]Ga-NYM046	^18^F-FDG	*z*	*P*	[^68^Ga] Ga-NYM046	^18^F-FDG	χ^2^	*P*
Primary tumor (*n* = 9)	13.5 (7.2, 16.2)	2.4 (2.2, 2.6)	−2.668	0.008	9	1	6.125	0.008
Lymph node metastases (*n* = 96)	5.9 (4.8, 7.8)	7.6 (5.0, 9.4)	−3.236	0.001	95	92	0.800	0.375
Distant metastases (*n* = 147)	5.0 (3.8, 7.1)	5.0 (3.7, 6.8)	−0.381	0.703	137	127	4.050	0.041

SUV_max_ is expressed as median followed by first and third quartiles in parentheses and was assessed using nonparametric Wilcoxon signed-rank test. Lesion numbers were assessed using McNemar test.

The uptake of [^68^Ga]Ga-NYM046 and ^18^F-FDG in all tumor lesions was further analyzed (Table 2). In primary tumors, the SUV_max_ for [^68^Ga]Ga-NYM046 was significantly higher than that for ^18^F-FDG (median, 13.5 vs. 2.4; *z* = −2.668, *P* = 0.008). However, a lower SUV_max_ was observed for [^68^Ga]Ga-NYM046 PET/CT than for ^18^F-FDG PET/CT for metastatic lymph nodes (median, 5.9 vs. 7.6; *z* = −3.236, *P* = 0.001). Moreover, no significant differences in SUV_max_ were found between the 2 agents for distant metastases (median, 5.0 vs. 5.0; *z* = −0.381, *P* = 0.703).

**FIGURE 6.**
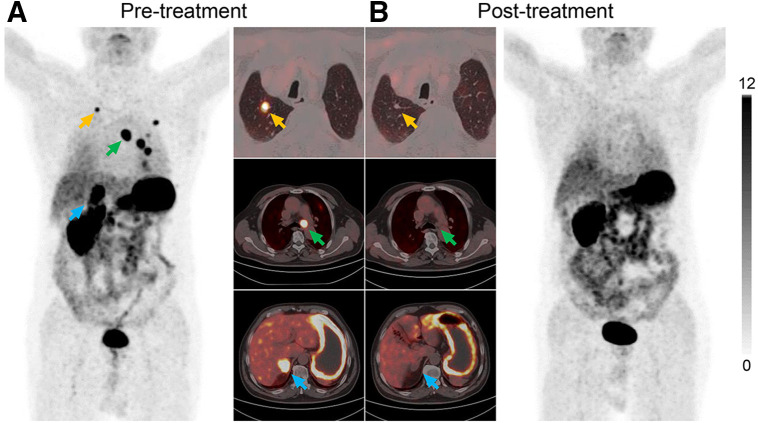
Images of patient 12 (73-y-old man), who underwent left nephrectomy with pathologically confirmed ccRCC and suspected tumor metastases on follow-up. (A) Images from pretreatment [^68^Ga]Ga-NYM046 PET/CT demonstrate mediastinal lymph node metastases (green arrows), lung metastases (yellow arrows), and right adrenal metastases (blue arrows). (B) After 2 treatment cycles, all metastases were negative for uptake.

Patient 12, a 73-y-old man, had undergone a total right nephrectomy several years previously and was pathologically diagnosed with ccRCC (Fig. 6). During follow-up, lesions in the right adrenal gland, multiple pulmonary nodules, and enlarged mediastinal lymph nodes were identified. [^68^Ga]Ga-NYM046 PET/CT confirmed that CAIX was highly expressed at all these sites, with an SUV_max_ of 23.9. After 2 cycles of treatment with a programmed death receptor 1 inhibitor and a protein tyrosine kinase inhibitor, posttreatment [^68^Ga]Ga-NYM046 PET/CT showed partial shrinkage of the lesions compared with the previous CT images, as well as a significant decrease in uptake on PET.

Some patients in the study underwent nephrectomy after PET/CT, and our group obtained 6 tumor section specimens for immunohistochemical studies with the patients’ informed consent. The immunohistochemical staining results revealed remarkable CAIX expression in these tumors (Supplemental Fig. 6A). Quantitative analysis showed that the higher [^68^Ga]Ga-NYM046 uptake in primary renal tumors corresponded to higher CAIX expression, with an *R*^2^ value of 0.8274 (Supplemental Fig. 6B).

## DISCUSSION

Renal cell carcinoma is the most common type of kidney cancer (21). Despite improvements in the 5-y survival rate, the prognosis for patients with advanced ccRCC remains unfavorable (22). Over the past 2 decades, there has been a significant evolution in the diagnosis and treatment of ccRCC, transitioning from traditional to innovative targeted and immunotherapeutic strategies.

Recently, Wu et al. created a novel CD70-targeted tracer, [^18^F]RCCB6, highlighting the utility of immuno-PET/CT for assessing tumor burden and monitoring treatment responses in patients with advanced ccRCC (23,24). In addition to CD70, CAIX also has demonstrated promising efficacy in theranostics for ccRCC. For example, Turkbey et al. conducted a phase II pilot study of [^18^F]F-VM4–037 and found that the tracer posed challenges in evaluating primary ccRCC lesions (25). Kulterer et al. studied the performance of [^99m^Tc]Tc-PHC-102, administering a high dose of the tracer to each patient (600–800 MBq) (26).

In this study, we developed the radiotracer [^68^Ga]Ga–NYM046, characterized by satisfactory purity and stability. Compared with NY104, which also features acetazolamide as its base structure, NYM046 shows similar CAIX-targeting affinity (4.98 nM vs. 5.75 nM) and reduced uptake in normal tissues, including kidney, lung, and bone, as observed in animal models (27). In patients, [^68^Ga]Ga-NYM046 PET/CT detected all primary tumors (9/9), whereas [^68^Ga]Ga-NY104 PET/CT showed an accuracy of only 58% (11/19) in cases of a primary renal mass (28). The main structural distinctions between NY104 and NYM046 are the incorporation of a DOTA macrocycle and a naphthyl group. DOTA and its derivatives are known to form highly stable complexes with ^68^Ga (29,30). Furthermore, previous studies on PSMA-617 have shown that incorporating a naphthylic linker significantly enhances tumor targeting and biologic activity and could help to optimize pharmacokinetics and improve imaging contrast (31,32). In the blocking assay, unlabeled NYM046 effectively inhibited binding of [^68^Ga]Ga-NYM046 to CAIX, further confirming its targeting specificity.

In the clinical study, [^68^Ga]Ga-NYM046 was highly concentrated in the kidneys because of eventual excretion through the urinary system, and it showed high accumulation in the stomach because of the physiologically high expression of CAIX in the human gastric mucosa (33). In a lesion-based analysis, [^68^Ga]Ga-NYM046 PET/CT detected more primary and metastatic lesions of ccRCC than did conventional CT or ^18^F-FDG PET/CT. [^68^Ga]Ga-NYM046 uptake was typically higher than ^18^F-FDG uptake in primary tumors but was lower in involved lymph nodes and comparable in distant metastases. One reason is that immune cells are an important component of the tumor microenvironment and are present at all stages of tumorigenesis (34). Particularly in ^18^F-FDG PET/CT, inflammation and the associated reactive activation of tissues can lead to nonspecific uptake in immune cells (35).

Furthermore, this study revealed a positive correlation between the expression of CAIX and the SUV_max_ of [^68^Ga]Ga-NYM046. However, the regulation of CAIX expression is predominantly governed by HIF-α–mediated mechanisms. Considering that hypoxia often occurs in solid tumors (36,37), it is important to be aware of the risk for secondary malignancies, with prostate, breast, colon, bladder cancers, and non-Hodgkin lymphoma being the 5 most common secondary malignancies in certain individuals with ccRCC (38). From another perspective, this insight suggests that tracers targeting CAIX may offer a broader scope of clinical utility, extending beyond the traditional association with ccRCC (Supplemental Fig. 7). Further research will be necessary to explore this potential.

The field of integrated diagnostic and therapeutic tracers is gaining increasing prominence. Radiopharmaceutical therapy with [^177^Lu]Lu-PSMA-617, used in patients with PSMA-positive metastatic castration-resistant prostate cancer, has shown efficacy in prolonging progression-free and overall survival (39). Consequently, NYM046, which contains a DOTA structure for stable binding with ^177^Lu, holds certain potential for radiopharmaceutical therapy. However, the aggregation of NYM046 in the gastric mucosa may raise concerns about potential normal-tissue injury and warrants further investigation in future studies.

This study had some limitations. First, the study’s small sample of patients may introduce statistical bias. Second, because of the potential side effects of invasive examinations, the metastatic lesions in the patients were not pathologically confirmed, potentially leading to diagnostic errors. We anticipate addressing these issues in future research.

## CONCLUSION

We successfully developed a CAIX-targeting small-molecule tracer, [^68^Ga]Ga-NYM046, that might offer a practical approach for diagnosing and monitoring treatment efficacy in patients with ccRCC.

## Notes

KEY POINTS **QUESTION:** Does [^68^Ga]Ga-NYM046, a novel CAIX-targeted PET tracer, perform better than ^18^F-FDG in the diagnosis of ccRCC? **PERTINENT FINDINGS:** In a prospective study of 12 patients with ccRCC, [^68^Ga]Ga-NYM046 PET/CT was superior to ^18^F-FDG PET/CT in the diagnosis of primary tumors and comparable in the detection of metastatic lesions. **IMPLICATIONS FOR PATIENT CARE:** [^68^Ga]Ga-NYM046 provides a promising method for diagnosing and monitoring treatment efficacy in patients with ccRCC.
